# Calcineurin controls the cytokinesis machinery during thermal stress in *Cryptococcus deneoformans*

**DOI:** 10.1101/2025.02.17.638697

**Published:** 2025-04-08

**Authors:** Vikas Yadav, Anna Floyd Averette, Rajendra Upadhya, Joseph Heitman

## Abstract

Calcineurin is a highly conserved phosphatase that plays a central role in sensing calcium and governing transcriptional, post-transcriptional, and post-translational signaling networks. Calcineurin is a heterodimer consisting of a catalytic A subunit and a regulatory B subunit. Through downstream effectors, calcineurin signaling drives myriad responses in different organisms. In the fungal pathogenic *Cryptococcus* species complex that infects humans, calcineurin governs thermotolerance and is essential for growth at high temperature and pathogenesis. In *Cryptococcus deneoformans* , the underlying molecular functions of this critical signaling cascade are not well understood. In this study, we conducted a genetic screen and identified genetic changes that suppress the requirement for calcineurin during high-temperature growth. Our results identified two mechanisms that bypass the requirement for calcineurin function. The first mechanism involves segmental aneuploidy via both amplification as well as loss of chromosome fragments. The second mechanism involves dominant amino acid substitution mutations in the genes encoding three proteins, Chs6, Imp2, and Cts1, orthologs of components of the Ingression Progression Complex required for septation and budding in *Saccharomyces cerevisiae* . Loss of calcineurin activity causes chitin and chitosan accumulation and severe budding defects, whereas suppressor mutations largely restore growth and cytokinesis in the absence of calcineurin. These findings reveal the calcineurin signaling cascade controls a conserved cytokinesis machinery at the mitotic exit network during thermal stress.

**Significance Statement:**

Cellular responses to external stress conditions involve activation of signaling cascades, which act via effectors to govern key cellular processes. One such signaling cascade involves the phosphatase calcineurin, the conserved target of cyclosporin A and FK506 that governs myriad cellular functions through its substrates. In human fungal pathogens, calcineurin is critical for pathogenicity. Here, we employed a genetic screen to map the functional interactions of this signaling cascade and repeatedly identified mutations in components of a single protein complex, suggesting a focused downstream response. With genetic and biochemical approaches, we established that these proteins play a crucial role in cell division at high temperature enabling cell division and revealing new avenues of research that could identify potential antifungal drug targets.

## Full Text Availability

The license terms selected by the author(s) for this preprint version do not permit archiving in PMC. The full text is available from the preprint server.

